# Social Media as a Surveillance Tool for Monitoring of Isotretinoin Adverse Effects

**DOI:** 10.7759/cureus.10327

**Published:** 2020-09-09

**Authors:** Saira E Alex, Christopher Wong, Alay Shah, Pooja Reddy, Logan DeBord, Harry Dao

**Affiliations:** 1 Dermatology, Baylor College of Medicine, Houston, USA; 2 Dermatology, Johns Hopkins University School of Medicine, Baltimore, USA; 3 Dermatology, University of Colorado School of Medicine, Presbyterian/St. Luke’s Hospital, Denver, USA; 4 Dermatology, Loma Linda University Health, Loma Linda, USA

**Keywords:** isotretinoin, acne, social media, instagram, accutane

## Abstract

Social media is an underutilized method for the surveillance of the patient perspective regarding their pharmacologic therapies. The purpose of this study was to investigate the nature of content posted on the social media platform Instagram with respect to the systemic acne medication isotretinoin.

The search term “#accutane” was queried into Instagram to generate all public posts using this hashtag between February 1 and May 31, 2018. Four independent investigators then scrutinized posts for eligibility. Our inclusion criteria were posts written in English, accessible by URL, primarily focused on isotretinoin, and posted by users of the medication. Data regarding multiple variables (tone of post, reason for positive or negative elements, posting of a face or other body part, mention of side effects, etc.) from each individual post was then entered into a Microsoft Excel template.

Of 7,661 posts, 3,082 were eligible. Among posts that contained negative tone (n=1312), this element was more commonly due to the presence of side effects (65%) than lack of improvement in skin appearance (33%). Overall, 1,263 posters (41%) mentioned adverse effects of oral isotretinoin, most commonly dry facial skin (17%), dry/cracked lips (16%), or arthralgias/myalgias (8%). Neuropsychiatric side effects were also documented, with users reporting fatigue (4%), mood changes (3%), and headache (2%).

In conclusion, reported side effects of oral isotretinoin on Instagram closely tracked its known side effects in frequency. Social media may be a valuable tool to surveil the general pattern and burden of adverse effects for patients undergoing treatment of dermatologic conditions.

## Introduction

Acne vulgaris is a common dermatological disease of the pilosebaceous unit due to an overproduction of sebum. In 2013, 5.1 million Americans sought treatment for acne, which resulted in $398 million of lost productivity for patients and caregivers. Affected patients frequently suffer from pain, erythema, swelling, and dryness in affected areas, and face an increased risk of permanent scarring and hyperpigmentation [1]. For patients with scar formation significant enough to warrant systemic therapy or for those who have failed other treatments, isotretinoin is the first-line option [2]. The current understanding of isotretinoin usage, however, would benefit from more frequent monitoring of skin improvement and side effect manifestations (e.g. daily rather than monthly reports), as well as from investigation of patients’ attitudes toward this high-risk, high-reward medication.

Social media platforms like Instagram allow users to share their own perspective on activities of daily living, including personal hygiene. Consumers can use this platform to share their life journey, and they use built-in features like hashtags and emojis as expressive tools. A hashtag (#) is followed by a relevant word or phrase, and can increase the visibility of a public post. Emojis are ideograms used to convey tone and mood. The platform records the number of likes for both photos and videos to highlight the popularity of a post, and it also records view counts for videos to highlight the public reach of the post. 

With Instagram’s minimalist style and emphasis on visuals, some users have been encouraged to track their skin improvement and report their opinions of various skin care products for their followers. Instagram has also been shown to be a useful tool in monitoring drug interactions and adverse drug reactions due to the large amount of data it contains; a 2016 study illustrated the utility of Instagram in associating a psoriasiform dermatosis with both metformin and verapamil [3]. Social media users have various reasons for choosing Instagram, but the most common reason found in a 2017 survey was a desire to belong [4]. This, combined with Instagram’s intrinsic ability to help its users expand their horizons, make connections, and share personal content, allows conditions like acne to become popular subjects of discussion in the online community. 

Instagram is poised to serve as a forum for users to share and receive tips to alleviate the burden of acne. While acne has been studied extensively with regards to its epidemiology and pathophysiology, a better patient-centered understanding of its most effective pharmacological treatment, isotretinoin, could be of use; our study investigates the nature of isotretinoin-related content posted on the social media platform Instagram.

## Materials and methods

Inclusion and exclusion criteria

We included posts (1) captioned with #accutane, (2) written in English, (3) accessible by URL, and (4) posted publicly between February 1, 2018 and May 31, 2018. We excluded posts (1) not written in English, (2) with a broken URL, or (3) not focused on isotretinoin, a different product, or a clinic.

Categorization of data

Four medical students independently assessed the posts for eligibility. The primary outcome variable studied was the content of the posts, analyzed using a categorical binary scoring system delineated in a similar study [5]. This included: media format (picture or video), number of likes, user habits (use of other products concurrently, on an ‘as needed’ basis, not completing prescribed course), tone of post (mostly positive, mostly negative, mostly mixed, or neutral), temporal relation of posting to treatment (pre-, during, post-treatment, or combination of pre-, during, or post-treatment), and side effects (28 possibilities). The tone of the posts were judged by the panel of four medical students to be positive, negative, mixed, or neutral based on the overall impression of the diction, syntax, and emojis used in the caption. Words conveying “happy,” “progress,” “relieved,” “optimistic,” “disappointed,” and “painful” were indicators of the user’s tone. Of note, ‘tone of post’ was later re-categorized to ‘user satisfaction’ where mostly positive tone was classified as ‘satisfied,’ mostly negative as ‘dissatisfied,’ and mostly mixed and neutral grouped into ‘neither satisfied nor dissatisfied.’

## Results

**Figure 1:**
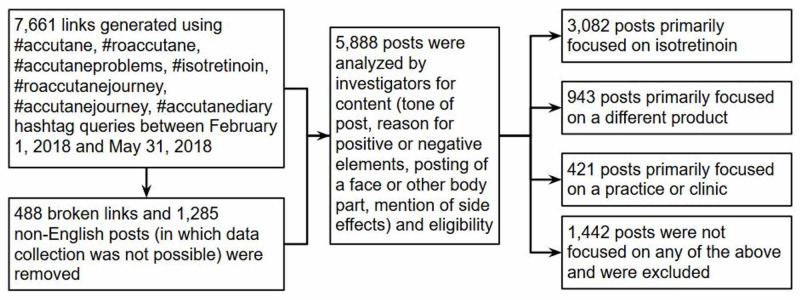
Flowchart describing the inclusion/exclusion and categorization of Instagram posts containing the #accutane hashtag between the period February 1, 2018 and May 31, 2018.

By querying the #accutane hashtag, we generated 7,661 links to Instagram posts for the period between February 1, 2018 and May 31, 2018. Of these, analysis was not possible for 488 non-functional links and 1,285 non-English language posts, which were thus excluded. We identified 3,082 posts that primarily focused on a user’s personal experience with isotretinoin, 943 posts focused on one or more different products, and 421 posts focused on promotion of a clinic or practice. The remaining 1,442 posts were excluded from further analysis (Figure 1).

2,983 photo posts and 99 video posts were categorized as directly relating to a user’s personal experience with isotretinoin. The average number of likes for photos and videos were 75.69 and 46.44, respectively, and the median number of likes was closer at 34 for photos versus 44 for videos (Table 1).

**Table 1:** Post type (photo or video), average and median “likes.”

Post Identifiers	# of Posts
Photo	2,983
Average # likes	75.69
Median # likes	34
Video	99
Average # likes	46.44
Median # likes	44

The most common side effects explicitly stated in the captions of these 3082 posts were dermatological (dry skin, 17.3%; dry/chapped lips, 15.8%; erythema, 7.4%; itching, 3.3%; angular cheilosis, 1.1%) and musculoskeletal (arthralgias and myalgias, 7.9%). Other side effects included fatigue (4.3%), mood changes (3.0%), epistaxis (2.7%), headache (2.2%), photosensitivity (1.6%), hair changes (1.1%), gastrointestinal (0.4%), and nonspecific/unspecified (5.5%). Accounting for users that reported multiple side effects in a single post, a total of 1263 posts (41%) made mention of at least one side effect (Table 2).

**Table 2:** Proportion of Instagram posts with the #accutane hashtag between February 1, 2018 and May 31, 2018 with mention of isotretinoin-associated side effects.

Side Effects	# of Posts
Dry skin	533 (17.29%)
Dry/chapped lips	488 (15.83%)
Arthralgias, myalgias	242 (7.85%)
Erythema	228 (7.40%)
Fatigue, sleepiness	133 (4.32%)
Itching	103 (3.34%)
Dry eyes	93 (3.02%)
Mood changes	90 (2.92%)
Epistaxis	82 (2.66%)
Headache	69 (2.24%)
Photosensitivity	48 (1.56%)
Hair changes	35 (1.14%)
Angular cheilosis	33 (1.07%)
Gastrointestinal	13 (0.42%)
Other	169 (5.48%)
Any side effect	1,263 (40.98%)

With regards to the perceived tone of the 3,082 posts related to a user’s personal experience with isotretinoin, a plurality of 1,239 posts (40.2%) had a mostly positive tone. Posts that were determined to be mostly mixed, mostly negative, or neutral in tone made up 21.2%, 21.3%, and 15.2% of the total, respectively. Sixty-two posts (2.0%) were not able to be assessed for tone (Table 3). Spanning posts of all tones, 1,410 posts (45.8%) included mention of improvement in skin appearance, while 433 posts (14.1%) explicitly noted lack of improvement. About a quarter of analyzed posts (855 posts, 27.7%) identified isotretinoin-related side effects as a contributing factor to the user’s negative tone (Table 4).

**Table 3:** Perceived tone of posts. Tone of post was assessed independently by investigators using the previously described protocol to ensure inter-rater reliability.

Tone of Post	# of Posts
Mostly positive	1,239 (40.20%)
Mostly mixed	653 (21.19%)
Mostly negative	659 (21.38%)
Neutral	469 (15.22%)
Unable to assess tone	62 (2.01%)

**Table 4:** Rationale for positive or critical commentary. Tone of post was assessed independently by investigators using a standardized protocol to ensure inter-rater reliability.

Tone of Post	# of Posts
Positive elements due to improvement in skin appearance	1,410 (45.75%)
Negative elements due to lack of improvement	433 (14.05%)
Negative elements due to side effects	855 (27.74%)

We briefly analyzed the 943 posts that focused on products other than isotretinoin. The most common were other medicated acne products (373 posts, 39.55%), moisturizers (218 posts, 23.12%), cosmetics (162 posts, 17.18%), and lip products (104 posts, 11.03%) (Table 5).

**Table 5:** Primary focus of product-centered posts queried using #accutane hashtag.

Product-Centered Post Focus	# of Posts
Makeup/Cosmetics	162 (17.18%)
Moisturizer	218 (23.12%)
Lip Product	104 (11.03%)
Sunscreen	42 (4.45%)
Vitamins/Supplements	44 (4.67%)
Food	62 (6.57%)
Other medicated acne products	373 (39.55%)
Other	162 (17.18%)

## Discussion

Isotretinoin is one of the pharmacotherapies for the treatment of moderate to severe acne vulgaris [6]. Given the role of social media to inform public opinion in this era of medicine and pharmaceutical marketing, we aimed to gauge the online perception of isotretinoin and its side effects by investigating non-expert consumer drug testimonials on social media [7]. 

In addition to tone of posts, our study also analyzed side effects experienced by users of Instagram who record their experience online. As enumerated in Table 1, the most common adverse effects mentioned within the posts included in our study were dermatological and musculoskeletal. This does not represent the full adverse effect profile from case report findings, which include gastrointestinal, ophthalmological, psychiatric, dermatological, otolaryngological, musculoskeletal, and teratogenic side effects [8]. Taken together, this illustrates that a major limitation of side effect surveillance via social media is incomplete reporting by patients (or their failure to disclose). Dermatological or musculoskeletal side effects may have less associated stigma compared to those that are gastrointestinal or psychiatric in nature, affecting what users choose to share in a public, online forum.

A major limitation of our study is the singular focus on Instagram as representing all isotretinoin users. Applying our methodology to other social media sites such as Twitter, YouTube, and Facebook could potentially yield valuable insight about adverse effects experienced by isotretinoin users not on Instagram. Additionally, our study excluded non-English posts, which reduces the external validity of the adverse effect profile beyond English-speaking groups.

Interestingly, we also noted that some users used an #accutane hashtag as a marketing tool. Some companies used this hashtag to sell products that offer relief for the side effects of isotretinoin therapy. Out of the 5,888 posts analyzed, 943 posts (16%) had a primary focus on products, of which 218 posts (23%) advertised moisturizers, and 104 posts (11%) advertised lip ointments. Other companies capitalized on the popularity of this hashtag with Instagram users to promote alternative acne treatments, while others used this hashtag to market their product or their cosmetology practice. This finding underscores how the online dissemination of evidence-based medical information may be challenged by dishonest advertising by opportunistic individuals and businesses, who compete for users’ attention on social media and other Internet domains.

In order for dermatologists to better understand how cosmetic companies active on social media influence public perception, future studies could research companies advertising under #accutane, and review the types of products advertised and the evidence associated with the disseminated information. In the future, studies could also explore social media perception of antibiotics and painkillers. An Instagram search for #antibiotics generated 137,709 posts, and #painkillers generated 152,391 posts. Given the public health concerns associated with antibiotics and painkillers, these two classes of medications would be appropriate candidates for social media perception analyses.

## Conclusions

Social media remains an underutilized but promising source for surveilling the patient perspective on medical treatments, such as the perceived positive benefits and adverse effects of pharmacologic therapy. Our analysis of 3,082 Instagram posts with hashtags like #accutane revealed not only self-reported frequencies of the common side effects of isotretinoin, but also users’ positive or negative attitude toward the medication as attributed to the presence or absence of improvement in skin appearance and impact of adverse effects. We support further investigation into these online spaces to more adequately address patient concerns regarding high-risk medications such as isotretinoin. Furthermore, given the large volume of Instagram posts with the same hashtag that advertise alternative acne treatments or beauty products, we stress that healthcare professionals have a duty to dispel misleading or incorrect claims from individuals and businesses that primarily have a profit motive rather than prioritizing effective medical management. As social media becomes more omnipresent in our day-to-day activities and in medicine, further work should be directed towards determining how to best collect and analyze health information that is shared by patients on these platforms.
